# TFCP2 activates beta-catenin/TCF signaling in the progression of pancreatic cancer

**DOI:** 10.18632/oncotarget.19741

**Published:** 2017-07-31

**Authors:** Dai Yuedi, Cai Yuankun, Zhao Jiaying, Liu Han, Wang Yueqi, Liu Houbao, Zhang Dexiang

**Affiliations:** ^1^ Department of Medical Oncology, Cancer Hospital of Fudan University, Minhang Branch, Shanghai 200240, China; ^2^ General Surgery Department, The Fifth People’s Hospital of Shanghai, Fudan University, Shanghai 200240, China; ^3^ General Surgery Department, General Surgery Institute, Zhongshan Hospital, Fudan University, Shanghai 200032, China; ^4^ General Surgery Department, Zhongshan-Xuhui Hospital Affiliated to Fudan University, Shanghai 200031, China

**Keywords:** TFCP2, beta-catenin/TCF signaling, pancreatic cancer, oncogene

## Abstract

Aberrant activation of beta-catenin/TCF (T-cell factor) signaling is frequently observed in the pancreatic cancer. However, the regulation of nuclear beta-catenin/TCF transcription machinery remains largely unknown. In this study, TFCP2 (transcriptional factor CP2) expression in pancreatic cancer was detected by qPCR, immunohistochemistry and western blot. Western blot, colony formation assay, migration and invasion experiment were performed to investigate the effects of TFCP2 on the growth and migration of pancreatic cancer cells. *In vivo*, mouse metastasis models were utilized to determine metastasis ability. Western blots were used to evaluate the related protein expression. Luciferase reporter assay was used to explore the role of TFCP2 on beta-catenin/TCF signaling. We have shown that the transcription factor TFCP2 was up-regulated in the pancreatic cancer. Over-expression of TFCP2 promoted the growth, migration, invasion and colony formation of pancreatic cancer cells, while knocking down the expression of TFCP2 inhibited the growth, migration, invasion, colony formation and metastasis of pancreatic cancer cells. The mechanism study revealed that TFCP2 interacted beta-catenin, enhanced the interaction between beta-catenin and TCF4, and activated beta-catenin/TCF signaling. Taken together, our study demonstrated the oncogenic roles of TFCP2 in pancreatic cancer, and suggested that TFCP2 might be a target for the treatment of pancreatic cancer.

## INTRODUCTION

Beta-catenin/TCF signaling is crucial for the embryo development and tumorigenesis [1, 2]. Over-activation of beta-catenin/TCF signaling is very common in the progression of pancreatic cancer [3]. Understanding the regulation of beta-catenin/TCF signaling would benefit the therapy of pancreatic cancer.

Beta-catenin is the key molecule of beta-catenin/TCF signaling [1, 2]. Wnt ligand stimulation leads to the inactivation of cytoplasmic beta-catenin destruction complex, the translocation of beta-catenin from cytoplasm to nucleus and the nuclear accumulation of beta-catenin [4]. In the nucleus, beta-catenin formed a complex with TCF4 and other co-activators and activated the expression of numerous genes, such as Snail, C-myc and CyclinD1 [5]. Multiple regulators have been found for their roles in regulating the activation of membrane receptor Frizzle, the inactivation of beta-catenin destruction complex and the degradation of beta-catenin [6]. With regard to the regulation for the beta-catenin/TCF4 transcriptional machinery, several regulators have been found. ICAT has been identified for its ability to block the interaction between TCF4 and beta-catenin [7]. Also, Pygopus has been shown to bridge the interaction between beta-catenin/TCF complex and BCL9 [8]. However, the regulation for beta-cateni/TCF transcriptional machinery remains largely unknown.

TFCP2 was first identified as one of the risk factor for Alzheimer disease (AD) [9, 10]. Recently, TFCP2 has been demonstrated to be involved in several biological processes. TFCP2 has been reported to interact with Polycomb protein RNF2 and regulate the epigenetics [11]. In addition, TFCP2 has been shown to regulate the expression of male-deternine gene SRY [12]. Several studies have demonstrated the roles of TFCP2 in the tumorigenesis. The expression of TFCP2 has been reported as the oncogene and elevated in the hepatocellular carcinoma (HCC) and colon cancer [13–15]. TFCP2 promoted the carcinogenesis of HCC through different molecular mechanisms. For example, TFCP2 has been shown to transcriptionally up-regulate the expression of MMP9 [13, 16], and the small molecule inhibitor of TFCP2 abrogated HCC [17]. These findings indicated the oncogenic roles of TFCP2 in the tumorigenesis. However, the functions of TFCP2 in the pancreatic cancer remain unknown.

In this study, we examined the expression of TFCP2 in the pancreatic cancer, investigated its functions and explored the molecular mechanisms.

## RESULTS

### Elevated expression of TFCP2 was frequently observed in pancreatic cancer

**Figure 1:**
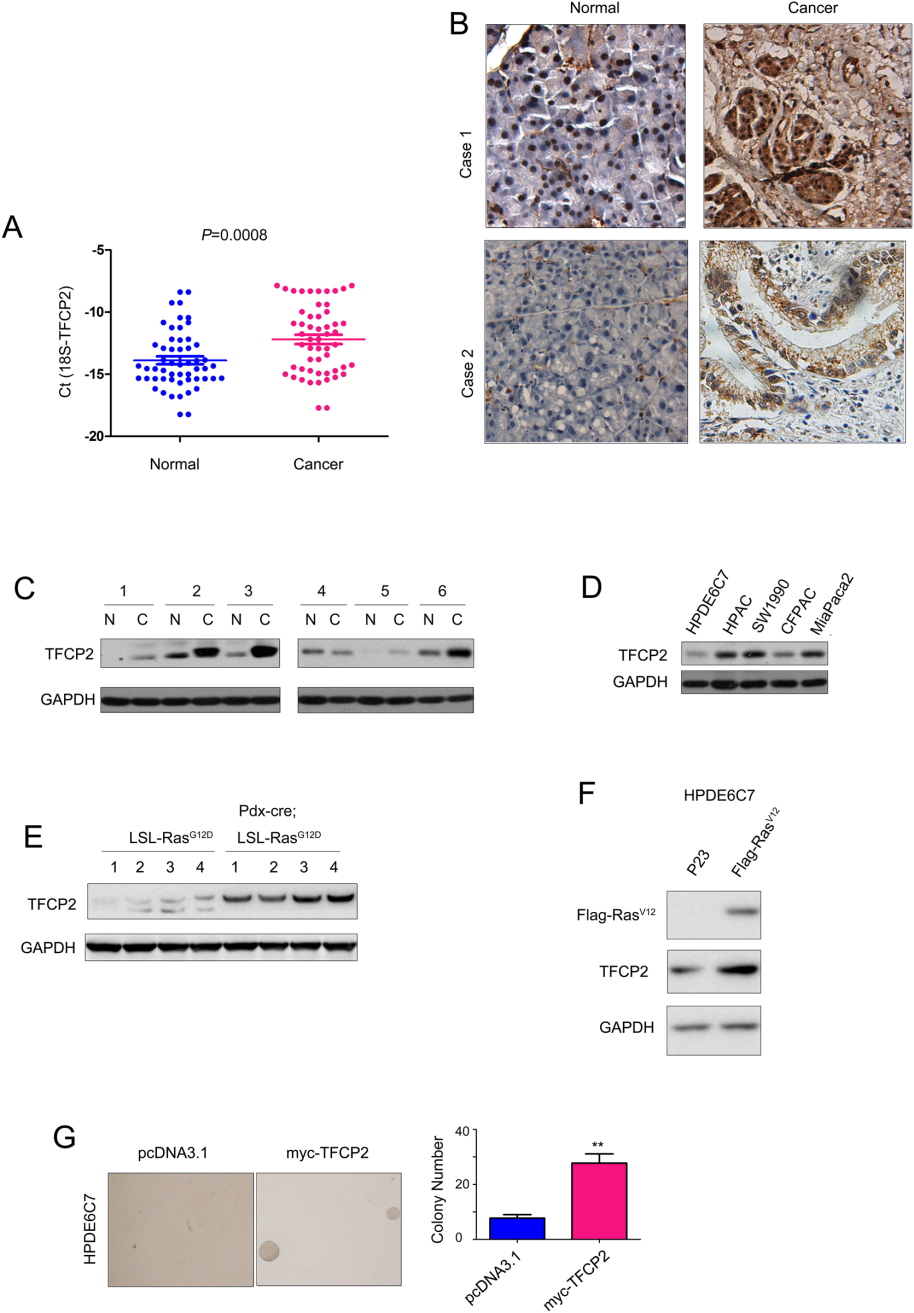
The expression of TFCP2 was elevated in pancreatic cancer **(A)** The mRNA level of TFCP2 was examined using q-PCR. The mRNA level of TFCP2 in 54 pancreatic cancer tissues and paired non-cancerous tissues was examined. 18S was used as the internal control. **(B)** The protein level of TFCP2 in two pancreatic cancer tissues was examined using IHC. **(C)** The protein level of TFCP2 in 6 pancreatic cancer tissues and paired normal tissues was examined using western blot. **(D)** The protein level of TFCP2 in normal pancreatic cells HPDE6C7 and pancreatic cancer cells was examined using western blot. **(E)** The protein level of TFCP2 in the pancreatic tissues of PDAC mouse model was examined using western blot. **(F)** The expression of oncogenic Ras elevated the protein level of TFCP2. **(G)** The effects of TFCP2 on the anchorage-independent growth of HPDE6C7 cells were evaluated using soft agar assay. **, *P*<0.01.

To study the expression pattern of TFCP2 in pancreatic cancer, we first examined the mRNA level of TFCP2 in 54 pancreatic cancer samples and paired adjacent normal tissues using q-PCR. Analyzing the q-PCR results revealed the significantly elevated expression of TFC2 mRNA in cancer tissues compared with the adjacent normal tissues (Figure 1A). Next, we examined the protein level of TFCP2 in pancreatic cancer by immunohistochemistry (IHC) and western blot. As shown in Figure 1B, increased protein level of TFCP2 was demonstrated by IHC staining, which was further confirmed by the findings shown in Figure 1C. In addition, we assessed the protein level of TFCP2 in a panel of pancreatic cancer cell lines (HPAC, CFPAC, SW1990 and MiaPaca-2) and normal pancreatic cells (HPDE6C7). Lower expression of TFCP2 was found in normal pancreatic cell line HPDE6C7 (Figure 1D). These observations suggested that the expression of TFCP2 was elevated in pancreatic cancer.

Next, we studied the expression of TFCP2 in the PDAC mouse model (Pdx-Cre; LSL-Ras^G12D^). The pancreatic tissues from the 8-month old mice were examined. Dramatic up-regulation of TFCP2 was observed in the PDAC mouse model compared with the control mice (Figure 1E), suggesting the expression of TFCP2 might be induced by oncogenic Ras sigaling. To test this hypothesis, oncogenic Ras (Flag-Ras^V12^) was over-expressed in HPDE6C7 cells and the protein level of TFCP2 was tested. It was found that the expression of TFCP2 was up-regulated upon the ectopic expression of Ras^V12^ (Figure 1F). Moreover, forced expression of TFCP2 promoted the malignant transformation of HPDE6C7 cells in the soft agar assay (Figure 1G). Taken together, these data suggested that TFCP2, induced by oncogenic Ras signaling, promoted the malignant transformation of normal pancreatic cells.

### TFCP2 promoted the growth, migration and invasion of pancreatic cancer cells

**Figure 2:**
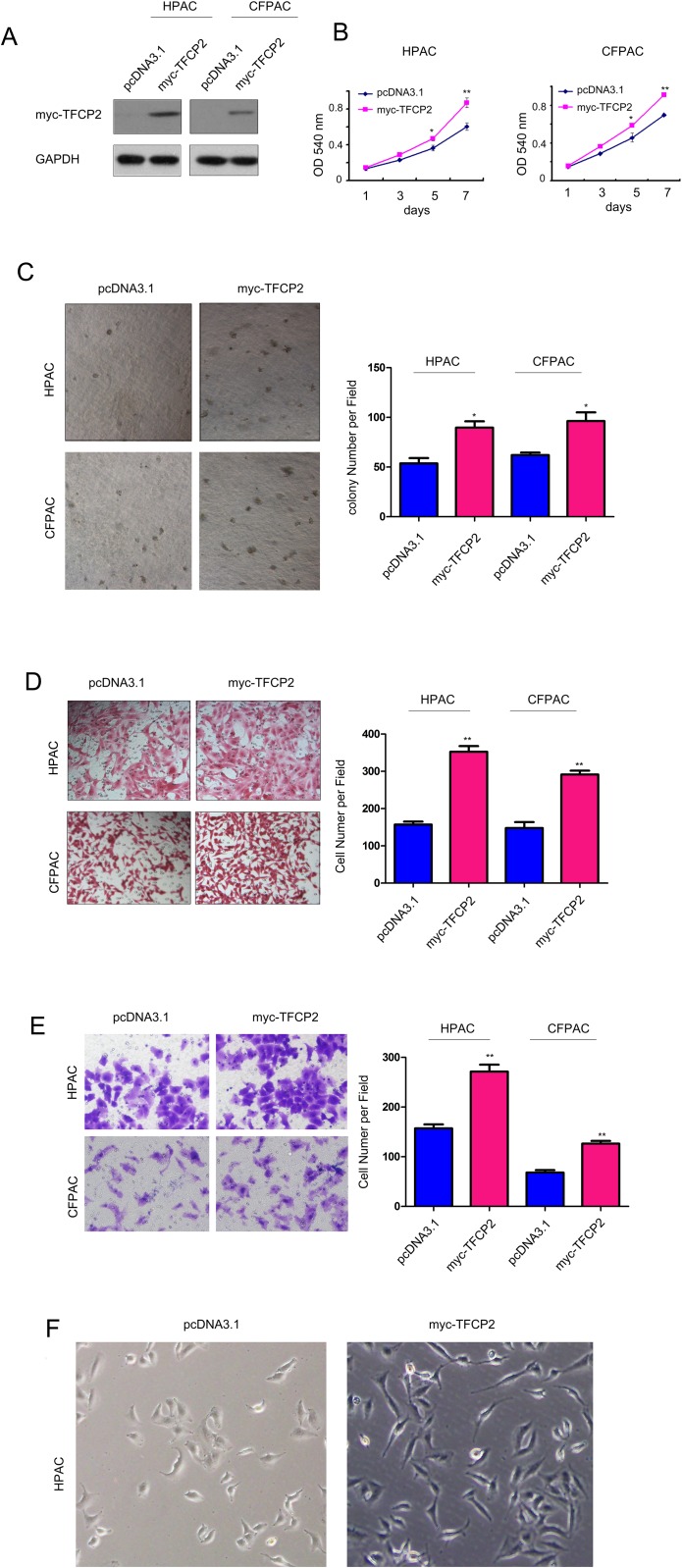
Overexpression of TFCP2 promoted the growth, migration and invasion of pancreatic cancer cells **(A)** Stable cell lines over-expressing myc-TFCP2 were established in HPAC and CFPAC cells. **(B)** MTT assay was used to evaluate the effects of TFCP2 on the growth of HPAC and CFPAC cells. **(C)** Soft agar assay was used to evaluate the effects of TFCP2 on the anchorage-independent growth of HPAC and CFPAC cells. **(D)** Boyden chamber assay was used to evaluate the effects of TFCP2 on the migration of HPAC and CFPAC cells. **(E)** Transwell assay was used to evaluate the effects of TFCP2 on the invasion of HPAC and CFPAC cells. *, *P*<0.05; **, *P*<0.01. **(F)** The morphology change of HPAC cell after up-regulating the expression of TFCP2.

To study the roles of TFCP2 in the progression of pancreatic cancer, we first over-expressed TFCP2 in HPAC and CFPAC cells (Figure 2A), and examined the effects of over-expressing of TFCP2 on the growth, migration and invasion of pancreatic cancer cells. MTT assay showed that over-expressing of TFCP2 promoted the growth of HPAC and CFPAC cells in the liquid culture as well as in the soft agar (Figure 2B-2C). Next, we examined the effects of TFCP2 on the motility of pancreatic cancer cells. As shown in Figure 2D, up-regulation of TFCP2 promoted the migration of HPAC and CFPAC cells. Moreover, up-regulation of TFCP2 enhanced the invasion of HPAC and CFPAC cells in the transwell assay (Figure 2E), and induced the morphology change of HPAC cells (Figure 2F).

**Figure 3:**
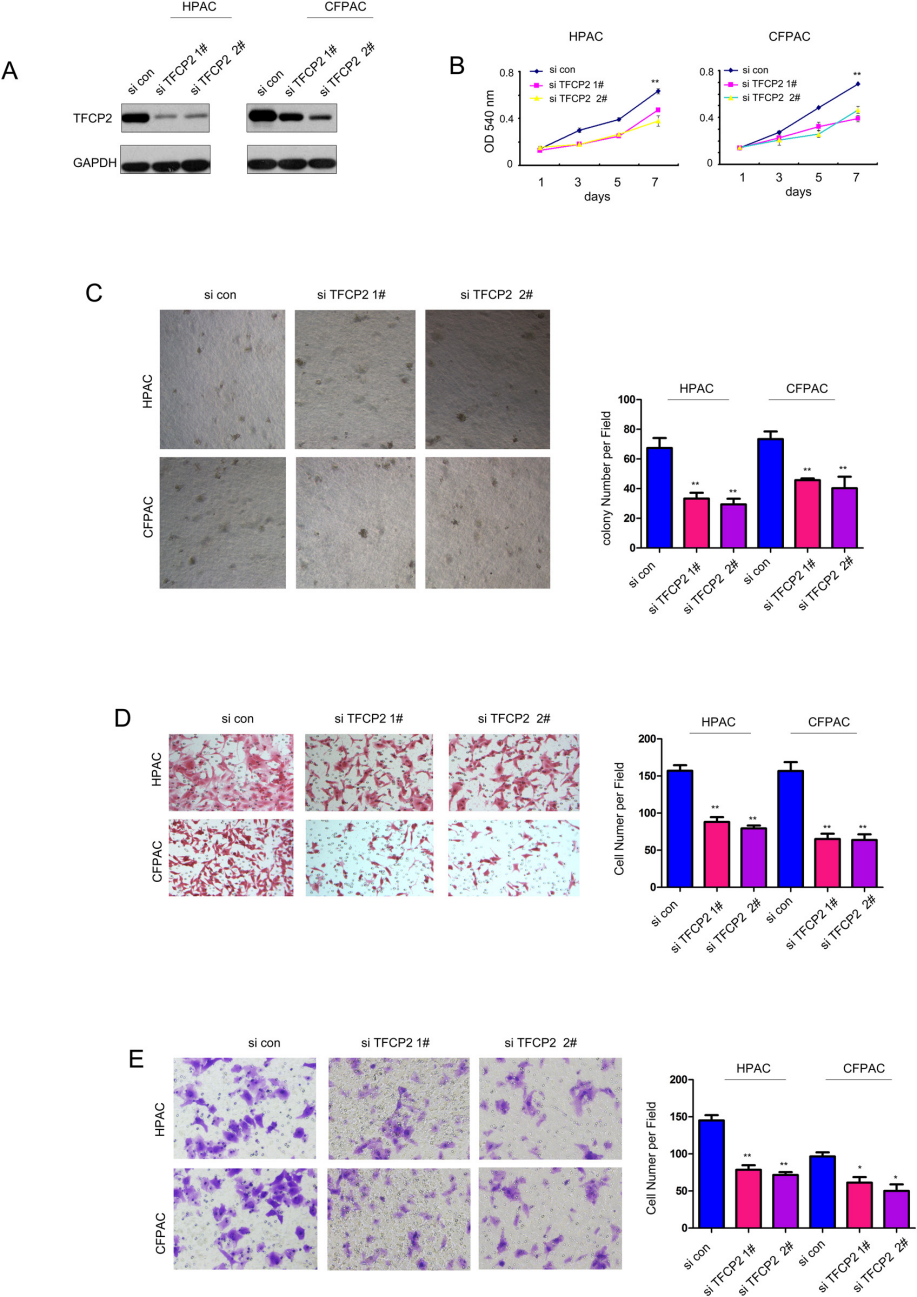
Knocking down the expression of TFCP2 inhibited the growth, migration and invasion of pancreatic cancer cells **(A)** The expression of TFCP2 was knocked down in HPAC and CFPAC cells. **(B)** MTT assay was used to evaluate the effects of down-regulating TFCP2 on the growth of HPAC and CFPAC cells. **(C)** Soft agar assay was used to evaluate the effects of down-regulating TFCP2 on the anchorage-independent growth of HPAC and CFPAC cells. **(D)** Boyden chamber assay was used to evaluate the effects of down-regulating TFCP2 on the migration of HPAC and CFPAC cells. **(E)** Transwell assay was used to evaluate the effects of down-regulating TFCP2 on the invasion of HPAC and CFPAC cells. *, *P*<0.05; **, *P*<0.01.

Next, we knocked down the expression of TFCP2 in pancreatic cancer cells (Figure 3A). Knocking down the expression of TFCP2 inhibited the growth of HPAC and CFPAC cells both in liquid culture and soft agar (Figure 3B-3C). Moreover, down-regulation of TFCP2 impaired the migration and invasion of the pancreatic cancer cells (Figure 3D-3E). Collectively, these results suggested that TFCP2 promoted the growth, migration and invasion of pancreatic cancer cells.

### TFCP2 promoted the growth, migration and invasion of pancreatic cancer cells by activating beta-catenin/TCF signaling

**Figure 4:**
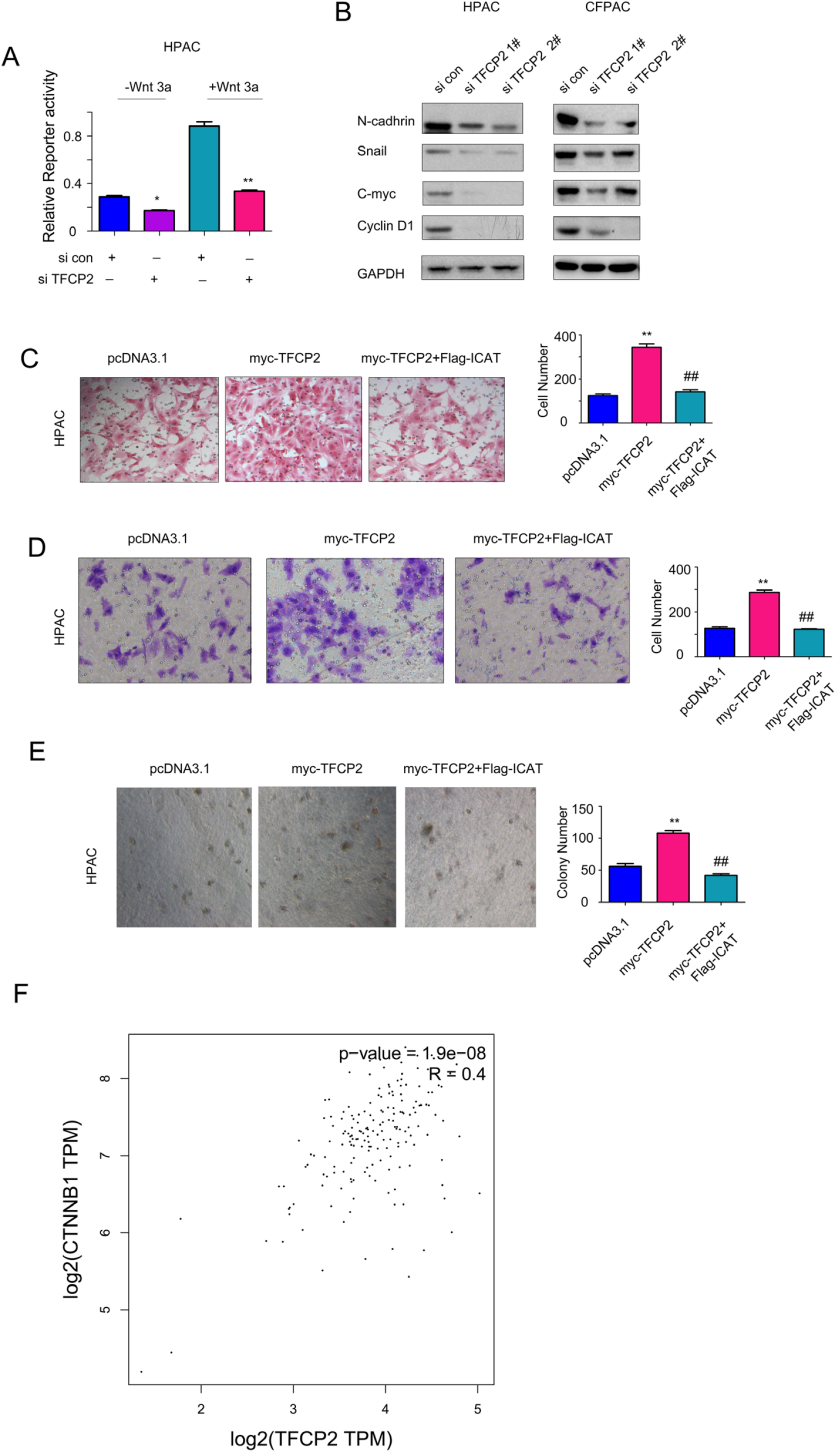
TFCP2 activated beta-catenin/TCF signaling in pancreatic cancer cells **(A)** The effects of TFCP2 expression on the activity of Topflash reporter were measured. **(B)** The effects of down-regulating TFCP2 on the expression of N-cadherin, c-Myc, CyclinD1 and Snail in HPAC cells were examined using western blot. **(C)** Over-expression of ICAT abolished the promoting-effects of TFCP2 on the migration of HPAC cells. **(D)** Over-expression of ICAT abolished the promoting-effects of TFCP2 on the invasion of HPAC cells. **(E)** Over-expression of ICAT abolished the promoting-effects of TFCP2 on the anchorage-independent growth of HPAC cells. **(F)** The positive correlation between TFCP2 and beta-catenin in pancreatic cancer. Over-expression *, *P*<0.05; **, *P*<0.01.

To understand the molecular mechanism through which TFCP2 positively regulated the growth, migration and invasion of pancreatic cancer cells, we screened the pathways regulated by TFCP2 using the reporter assay. In the screening, knocking down the expression of TFCP2 inhibited the activity of Topflash, an indicator for beta-catenin/TCF signaling, both at the base level and upon the treatment of wnt3a (Figure 4A). In addition, knocking down TFCP2 down-regulated the expression of several beta-catenin/TCF target genes, such as N-cadherin, Snail, C-myc and Cyclin D1 (Figure 4B). These data suggested that knocking down TFCP2 inhibited beta-catenin/TCF signaling. Futhermore, we examined whether TFCP2 promoted the growth, migration and invasion of pancreatic cancer cells by activating beta-catenin/TCF signaling. As shown in Figure 4C-4E, ICAT, the negative regulator for beta-catenin/TCF signaling, abolished the promoting effects of TFCP2 on the migration, invasion and anchorage-independent growth of HPAC cells. Moreover, in the GEPIA database, the expression of TFCP2 positively correlated with the expression of beta-catenin (Figure 4F). In summary, these findings suggested that TFCP2 promoted the growth, migration and invasion of pancreatic cancer cells by activating beta-catenin/TCF signaling.

### TFCP2 interacted with beta-catenin in pancreatic cancer cells

**Figure 5:**
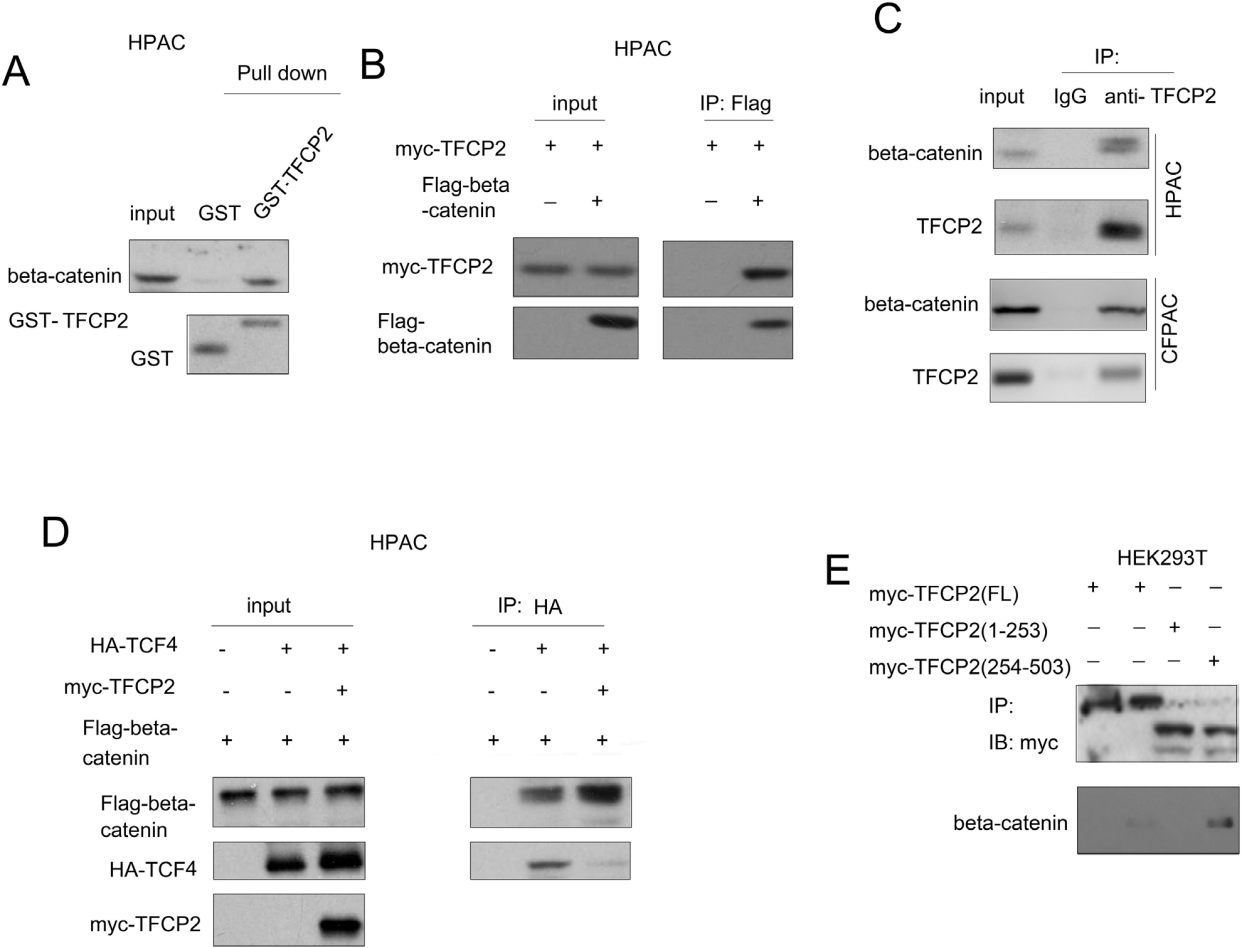
TFCP2 interacted with beta-catenin in pancreatic cancer cells **(A)** The interaction between beta-catenin and TFCP2 was examined using GST pull-down assay. **(B)** The interaction between ectopically expressed beta-catenin (Flag-beta-catenin) and TFCP2 (myc-TCP2) was examined using immunoprecipitation assay. **(C)** The interaction between endogenous beta-catenin and TFCP2 was examined using immunoprecipitation assay in HPAC and CFPAC cells. **(D)** The effects of TFCP2 expression on the interaction between beta-catenin and TCF4 was examined using immunoprecipitation assay. **(E)** Mapping the domains of TFCP2 for the binding of beta-catenin. Different TFCP2 plasmids were transfected into 293T cells. 48 hour later, the immunoprecipitation assay was performed using anti-myc antibody.

The activation of beta-catenin/TCF signaling promoted us to investigate whether TFCP2 interacted with beta-catenin/TCF transcription machinery. The Figure 5A showed that the fusion protein GST-TFCP2 directly interacted with beta-catenin in HPAC cells. Moreover, as shown in Figure 5B, ectopically expressed TFCP2 (myc-TFCP2) and beta-catenin (Flag-beta-catenin) interacted each other in HPAC cells. Furthermore, the endogenous TFCP2 and beta-catenin formed a complex (Figure 5C). These data demonstrated the interaction between beta-catenin and TFCP2. Next, we explored how the expression of TFCP2 activated beta-catenin/TCF signaling. It was found that the expression of TFCP2 enhanced the interaction between beta-catenin and TCF4 (Figure 5D). Morover, we mapped the domain in TFCP2 for the interaction of beta-catenin. It was found that the c-terminus of TFCP2 (254-503aa) was responsible for its interaction with beta-catenin (Figure 5E). Taken together, these findings suggested that TFCP2 activated beta-catenin/TCF signaling by bridging the interaction between beta-catenin and TCF4.

### Knocking down the expression of TFCP2 inhibited the metastasis of pancreatic cancer cells *in vivo*

**Figure 6:**
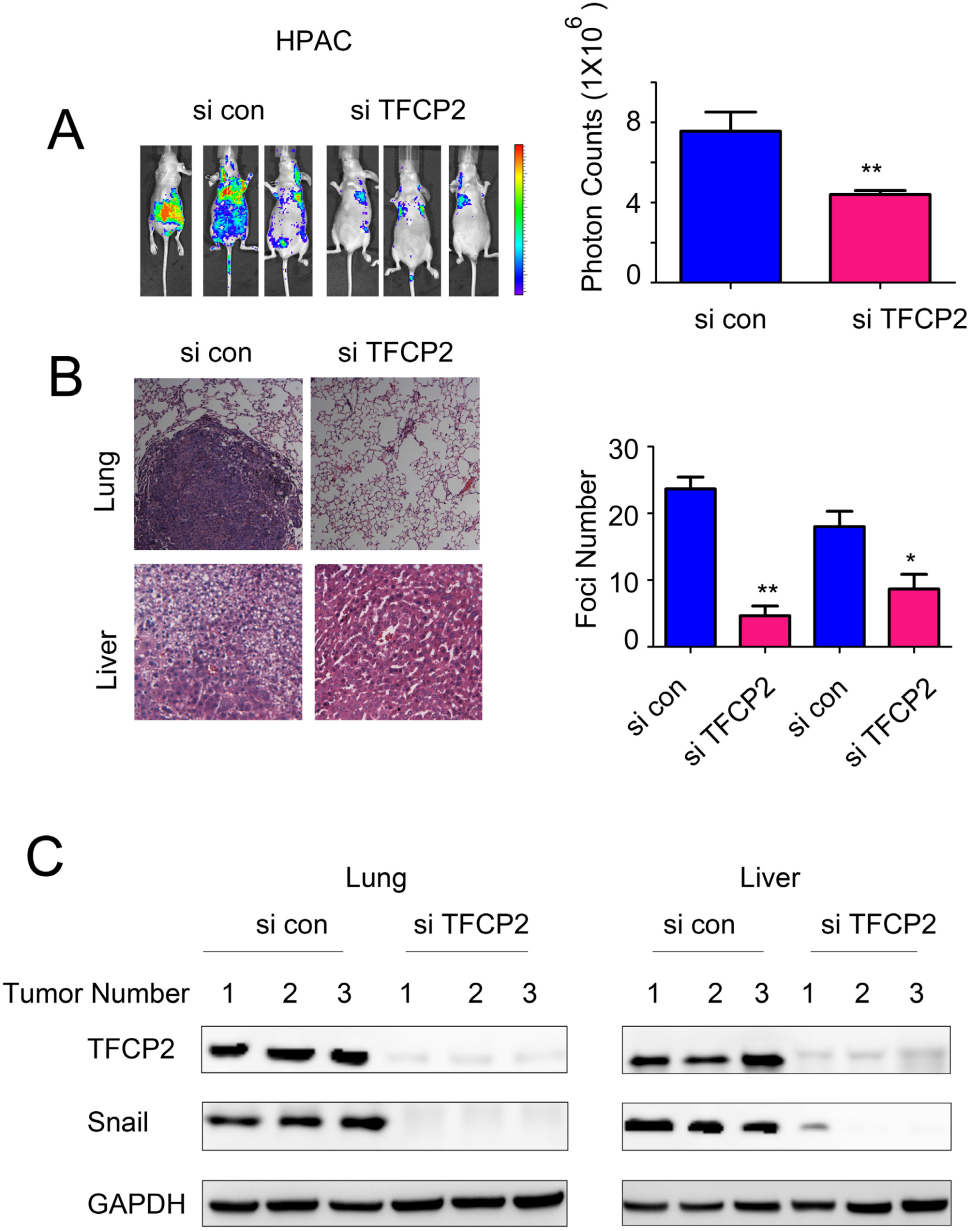
Knocking down the expression of TFCP2 inhibited the metastasis of HPAC cells *in vivo* **(A)** The *in vivo* image system was used to monitor the metastasis lesions formed by HPAC cells. **(B)** HE staining was used to examine the metastic foci formed in the lung and liver. *P*<0.05; **, *P*<0.01. **(C)** The knockdown efficiency and the expression of Snail in the tumors.

We next evaluated the functions of TFCP2 using an *in vivo* metastasis mouse model by injected the HPAC cells (labeled with luciferase gene) into the nude mice through the ventricle of the heart. The metastasis lesions were monitored using the *in vivo* imaging system. As shown in Figure 6A, down-regulation of TFCP2 inhibited the metastasis lesions number of HPAC cells *in vivo*. Moreover, knocking down the expression of TFCP2 inhibited the metastasis lesions in lung and liver (Figure 6B). Finally, we examined the knockdown efficiency of TFCP2 and the EMT-driver Snail in the metastatic tumors. It was found down-regulation of TFCP2 decreased the expression of Snail *in vivo* (Figure 6C). Collectively, these data suggested that knocking down the expression of TFCP2 inhibited the metastasis of pancreatic cancer cells *in vivo*.

## DISCUSSION

In this study, we have demonstrated that TFCP2, a transcription factor, was elevated in the pancreatic cancer. In the functional study, we have shown that TFCP2 promoted the transformation of normal pancreatic cells, promoted the growth, migration, invasion and metastasis of pancreatic cancer cells. The molecular mechanism investigations demonstrated the activation of beta-catenin/TCF signaling by TFCP2 in the pancreatic cancer cells. These findings supported the notion that TFCP2 is an oncogene in pancreatic cancer.

An interesting finding of this study is the up-regulation of TFCP2 in the pancreatic cancer. Previous studies have reported that the expression of TFCP2 was elevated in HCC and colon cancer [14, 15, 18]. The expression of TFCP2 was elevated in 90% HCC clinical samples, and its expression level showed significant correlation with the stages and grades of the disease. The functional study showed that forced expression of TFCP2 in less aggressive HCC cells resulted in highly aggressive, angiogenic and multi-organ metastatic tumors in nude mice. In addition, both TFCP2 mRNA and protein were up-regulated in colon cancer, and high TFCP2 expression correlated with large tumor size, advanced pN stage, advanced AJCC stage, high Ki-67 index and worse prognosis. However, the expression of TFCP2 was reported to be down-regulated in melanoma tissues, and over-expression of TFCP2 inhibited the anchorage-independent growth of melanoma cells through P21 pathway [18, 19]. These studies indicated that the functions of TFCP2 in the tumorigenesis were dependent on the tumor types and context.

Another important finding of this study is the induction of TFCP2 by the oncogenic Ras^V12^ signaling. The expression of TFCP2 in the normal pancreatic HPDE6C7 cells was dramatically increased upon the expression of TFCP2, and TFCP2 promoted the transformation of HPDE6C7 cells. These observations suggested that TFCP2 might mediate the oncogenic effects of Ras. It has been reported that TFCP2 was target of ERK, an effector of Ras [18]. Up to date, it is unsuccessful for the treatment of pancreatic cancer by directly targeting oncogenic Ras. This study indicated that it might be promising to target TFCP2 for the treatment of pancreatic cancer. Indeed, FQI1, the inhibitor for TFCP2 exhibited antiproliferative activity in multiple cancer cell lines, suggesting the potential clinical application of FOI1 for the treatment of pancreatic cancer.

The molecular mechanism study revealed the activation of beta-catenin/TCF signaling by TFCP2. TFCP2 strengthened the interaction between beta-catenin and TCF4. Previous reports showed that ICAT inhibited the interaction between beta-catenin and TCF4 [7, 20]. Consistently, in this study, it was found that over-expression of ICAT4 abrogated the promoting effects of TCP2 on the migration, invasion and anchorage-independent growth of pancreatic cells. Based on these observations, it would be hypothesized that in normal cells, TFCP2 competed with ICAT for binding beta-catenin and TCF4 and kept the interaction of beta-catenin and TCF4 in balance. In cancer cells, the balance was disrupted due to the up-regulation of TFCP2, which led to the activation of beta-catenin/TCF signaling.

In summary, our study demonstrated the oncogenic roles of TFCP2 in pancreatic cancer. Further study using the TFCP2 knock out mouse model would provide novel insight into the functions of TFCP2 in pancreatic cancer.

## MATERIALS AND METHODS

### Cell lines

Both pancreatic cancer cell lines (HPAC, CFPAC, MIAPaca2, SW1990 and PANC-1) and normal human pancreatic cell line (HPDE6C7) were obtained from ATCC (American Typical Culture Center). Cells were cultured in DMEM medium supplemented with 10% fetal bovine serum (GIBCO), 100units/mL penicillin and 100 μg/mL streptomycin in an incubator with 5% CO_2_ at 37°C.

### Ethics, consent and permissions

54 pancreatic cancer samples and paired normal tissues were collected from patients who subjected surgery atCancer Hospital of Fudan University and The Fifth People’s Hospital of Shanghai after obtaining the consent of the patients. Tissues were stored in liquid nitrogen. This study was performed after being approved by the ethics committee.

### PCR analysis

TRIzol was used to isolate total RNA from the clinical tissues. The reverse transcription kit was used to prepare the complementary DNA (cDNA). The expression of TFCP2 in the tissues was examined by quantitative real-time PCR using SYBR^®^ Green Realtime PCR Master Mix (TOYOBO) following the instructions of the manufacturer. Sequences of quantitative real-time PCR primers are listed as follows:

18S Forward primer: 5’-TAAATCAGTTATGGTTCCTT -3’.

18S Reverse primer: 5’-CGACTACCATCGAAAGTTGA-3’.

TFCP2 Forward primer: 5’-TGAGAATAAAATCCTGCCTT-3’.

TFCP2 Reverse primer: 5’-GCCATTAATTTCTGGAAGTT-3’.

### Western blot

The cellular proteins were resolved by SDS-PAGE after extracted by the RIPA buffer. The proteins were transferred to the PDVF membrane. After blocking with the 3% BSA solution, the membrane was incubated with following primary antibodies over night: anti-TFCP2 (Abcam), anti-Snail (Cell Signaling Technology), anti-Cyclin D1 and anti-c-Myc (Cell Signaling Technology), anti-GAPDH (Santa Cruz). The membranes were washed with TBST solution and incubated with the secondary antibody for 1 hour at the room temperature. The protein was visualized by ECL kit.

### Immunohistochemistry (IHC)

The sections were deparaffinized and rehydrated using xylene and ethanol. 0.35% H_2_O_2_ solution was used to block endogenous peroxidase activity. Antigens retrieve was performed using microwaving. Non-specific binding was blocked by 1% BSA solution Sections were stained with TFCP2 antibody and visualized with secondary antibody (Envision, Gene Techenology). Slides were then developed with DAB andcounterstained with hematoxylin.

### GST pull-down assay

The coding sequence of TFCP2 was cloned into the expression vector pGEX-4T-1. The fusion protein GST-TFCP2 was purified. The whole cell lysates of HPAC were prepared in 50 mM Tris-Cl (pH 7.5), 150 mM NaCl, 0.1% NP40 and protease inhibitor cocktail. 5 μg GST-TFCP2 fusion protein and 500 μg cell lysates were incubated at 4°C over night. 50 μl of glutathione-Sepharose-4B beads were added to the samples and incubated at 4°C for 1 hr to capture the GST fusion proteins. After washing with lysis buffer three times, the proteins were eluted in Laemmli buffer and analyzed by SDS-PAGE.

### Vector construction

The coding sequence of TFCP2 was amplified by PCR and inserted into the expression vector pcDNA3.1 to obtain the myc tagged TFCP2. The coding sequence of beta-catenin was amplified by PCR and inserted into the expression vector pCMVTag2B to obtain the Flag tagged beta-catenin. The coding sequence of TCF4 was amplified by PCR and inserted into the expression vector pCMV-HA to obtain the HA tagged TCF4.

### Knocking down the expression of TFCP2

RNAi lenti-virus particles (si con and si TFCP2) were purchased from GeneChem (China). The sequences for si TFCP2 were 5’-aagaagagtcgagtttgcctc-3’ and 5’-aaccatactcacagagtgttc-3’. The sequence for si con was 5’-ccaaaattcaccaggatctt-3’. Cells were infected with the indicated lenti-virus particles of the same MOI for 24 hours and then stable knock-down cells were selected with the medium containing puromycin for at least a week.

### Reporter assay

HPAC Cells were grown to a subconfluent density. 16 hours later, the reporter assays were performed using 0.1 μg of Topflash, 0.5 μg of expression vector, and 0.05 μg of TK Renilla luciferase (internal control for transfection efficiency). 48 hours later, cells were treated with Wnt3a protein for 8 hours. Then, cell lysates were prepared and the reporter activity was measured using the dual-luciferase reporter assay system (Promega).

### Migration assay

Boyden chamber was used to evaluate the motility of pancreatic cells. Cells (2×10^5^) suspended in 0.05ml medium containing 1% FBS were placed in the upper chamber, and the lower chamber was loaded with 0.152ml medium containing 10% FBS acting as the chemoattractant. 12 hours later, cells migrated to the lower surface of filters was detected with traditional hematoxylin and eosin (H&E) staining. The experiments were repeated for three times. Five random visual fields were counted for each sample and the average was determined.

### MTT assay

Cells were plated in 96-well plates at the density of 10^5^ cells/well. Cell growth was determined using the 3-(4, 5-methylthiazol-2-yl)-2, 5-diphenyltetrazolium bromide (MTT) colorimetric growth assay for a week. Every other day, cell growth was determined by adding MTT solution (50μg/well) for 4h. Cellular MTT was resolved with DMSO and was measured at 540 nm. All experiments were performed in triplicates.

### Soft agar assay

In soft agar assay, 5000 cells/well were suspended in the upper layer (0.35% agarose and 10% FBS in DMEM) in 6-well plates. The plates were coated with bottom layer (0.5% agarose and 10% FBS in DMEM). After 14 days of incubation, the colonies were counted and measured. All of the experiments were done at least three times.

### Mice model

Mice were housed and treated after being approved by the Institutional Animal Care and Use Committee of Fudan University. *Kras*^*G12D*^ and *Pdx-Cre* mice were obtained from Jackson Lab (Koch Institute for Integrative Cancer Research, Cambridge, MA). Pancreatic cancer mouse models *Pdx-Cre; Kras*^*G12D*^ mice were generated by crossing *Kras*^*G12D*^ and *Pdx-Cre* mice.

### Statistical analysis

Statistical analysis was performed by the Student *t*-test (two-tailed) using Prism GraphPad software. Differences with *P* < 0.05 were considered statistically significant. Data were represented as mean±SEM.

## Abbreviations

TFCP2: transcriptional factor CP2
TCF: T-cell factor
AD: Alzheimer disease
HCC: hepatocellular carcinoma
ATCC: American Typical Culture Center
IHC: immunohistochemistry
